# 5G AI-IoT System for Bird Species Monitoring and Song Classification

**DOI:** 10.3390/s24113687

**Published:** 2024-06-06

**Authors:** Jaume Segura-Garcia, Sean Sturley, Miguel Arevalillo-Herraez, Jose M. Alcaraz-Calero, Santiago Felici-Castell, Enrique A. Navarro-Camba

**Affiliations:** 1 Escola Tecnica Superior d’Enginyeria, Universitat de Valencia, 46100 Burjassot, Spain; miguel.arevalillo@uv.es (M.A.-H.); santiago.felici@uv.es (S.F.-C.); enrique.navarro@uv.es (E.A.N.-C.); 2 School of Computing, Engineering & Physical Sciences, University of the West of Scotland, Paisley PA1 2BE, Scotland, UK; sean.sturley@uws.ac.uk (S.S.); jose.alcaraz-calero@uws.ac.uk (J.M.A.-C.)

**Keywords:** AI-IoT, birdsong classification, CNN, audio

## Abstract

Identification of different species of animals has become an important issue in biology and ecology. Ornithology has made alliances with other disciplines in order to establish a set of methods that play an important role in the birds’ protection and the evaluation of the environmental quality of different ecosystems. In this case, the use of machine learning and deep learning techniques has produced big progress in birdsong identification. To make an approach from AI-IoT, we have used different approaches based on image feature comparison (through CNNs trained with Imagenet weights, such as EfficientNet or MobileNet) using the feature spectrogram for the birdsong, but also the use of the deep CNN (DCNN) has shown good performance for birdsong classification for reduction of the model size. A 5G IoT-based system for raw audio gathering has been developed, and different CNNs have been tested for bird identification from audio recordings. This comparison shows that Imagenet-weighted CNN shows a relatively high performance for most species, achieving 75% accuracy. However, this network contains a large number of parameters, leading to a less energy efficient inference. We have designed two DCNNs to reduce the amount of parameters, to keep the accuracy at a certain level, and to allow their integration into a small board computer (SBC) or a microcontroller unit (MCU).

## 1. Introduction

The assessment of environmental quality is a major issue in constructing an ecological ecosystem and is an important duty of all of society. In these ecosystems (whether urban or natural), birds play an essential role, and the formation of bird communities can be used as a parametric index of environmental quality [1].

The soundscape standard, ISO 12913 [2], in its second part, describes a taxonomy for sound classification, which takes into account the typology of the sound source and the environment, which is also specified in the taxonomy of the URBANSOUND datasets [3] but is orientated only to urban sounds [4]. The natural environment is also part of a more general vision of this taxonomy, as it considers other sounds apart from those produced in a human-centric vision.

In the natural environment, the exploration of avian life is of great importance in the realms of bird conservation, understanding the wetland ecosystem, and evaluating the quality of the ecosystem. As reported by the International Union for Conservation of Nature (IUCN), there are hundreds of bird species worldwide, a considerable percentage of them teetering on the brink of vulnerability and imminent extinction [5]. Due to birds’ remarkable traits, including exceptional mobility, extensive geographic range, and robust adaptability to diverse environments, their songs, serving as a telltale sign of their activities, are frequently harnessed for species detection, monitoring, and quantification. Birdsong exhibits rapid temporal variations, offering stability within the same species and clear distinctions between species. The development of automated bird classification models, using audio data derived from birdsong, holds immense promise for numerous applications in the realms of bird conservation, ecology, and data archiving.

In this context, the utilization of wireless acoustic sensor networks (WASN) can bring substantial value, as they can gather data and conduct measurements in a decentralized manner. In addition, they can be easily adjusted or reconfigured to accommodate additional measurements and incorporate new parameters. This network comprises multiple nodes, each equipped with its own processing unit, memory, and wireless communication modules, typically employing WiFi technology. These nodes are capable of connecting to microphones. Generally, these networks are cost-effective and offer the flexibility to expand the number of nodes according to specific requirements or testing scenarios. The nodes are typically built around versatile single board computers (SBC), such as the widely recognized Raspberry Pi (RPi) [6]. The nodes connected to the microphones can record audio with varying sampling frequencies and bit depths per sample. These networks find application in diverse tasks, including sound source localization [7], tracking of sound sources [8], sound source identification [9], and the measurement of specific environmental characteristics [10], among others.

The use of artificial intelligence (AI) combined with IoT systems has been applied in multiple fields. In [11], the authors develop an IoT system with AI capabilities to compute complex metrics (psychoacoustic parameters) for subjective annoyance assessment. This kind of development is orientated towards monitoring the environmental sound in a city. Other applications with a combination of AI and IoT systems can be seen in [12], where the authors use Raspberry Pi-based IoT systems to collect acoustic information from different rooms to determine the acoustic metrics of the room. This combination of technologies allows the design of cost-efficient monitoring systems. Furthermore, it has been applied to the areas of e-Health, in [13], where we can find a review of the applications of AI-IoT systems in the analysis, monitoring, and assessment during the COVID-19 pandemic. Another application orientated to Agriculture 5.0 is shown in [14], where the authors apply IoT and AI technologies to detect and control irrigation levels, plant diseases, and pest identification. In [15], the authors focus their efforts on applying AI and IoT for smart energy management in smart cities, so they explore the level of efficiency of ML/IoT techniques. Furthermore, it should be mentioned that the University of Cornell has developed an application for bird classification, based on the EfficientNetB0 neural network, named BirdNet [16], which is available for mobile phones and through the website.

This article explains the development of a 5G-IoT system to record birdsong and explores the birdsong classification based on [17], going beyond their results, by extending the study by collecting more species (up to 41 bird species) and comparing the raw dataset collected with a set combined with temporal augmentation techniques (i.e., noise addition, stretching, time shifting, pitch shifting) to provide a wider set of audio, and adding some post-processing techniques in the inference part to increase the percentage of accuracy in the detection. Furthermore, our objective is to improve the performance of the BirdNet application by training lighter neural networks to enable wider 5G IoT monitoring systems.

After this introductory section, the rest of the paper is structured as follows. Section 2 explains the materials and methods used in this investigation, describing the technologies and architecture used for the IoT system and the description of the dataset and the AI techniques (CNN-based) to compare. Section 3 shows the results obtained from the training of the selected set of CNNs for birdsong classification and the prediction done with the combination of the AI-IoT system with low-cost sensor and MCU (INMP441 [18] microphone and ESP32-S3 [19]). Finally, Section 4 concludes the paper, and some future work is defined.

## 2. Materials and Methods

In this section, we will provide information on the development of the IoT system architecture, which will collect audio information in the field. This system should provide efficient energy management to allow an extended period of data collection. Here, we use a node based on ESP32 microcontroller unit (MCU) architecture, as it has been proven [20] to be a cost-effective and powerful option (in the case of ESP32-S3 MCU) for audio collection and preprocessing [21].

### 2.1. IoT System Architecture

**Figure 1:**
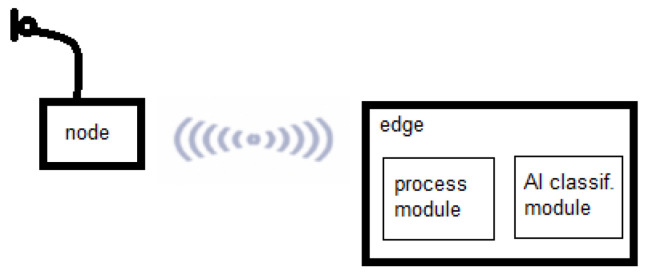
Schema of the AI-IoT system.

In previous work [22], a platform based on the ESP32 platform and LoRa was developed. In this case, we used a similar architecture but chose a newer version of the platform that had more RAM. Here, the IoT system is based on a ESP32-S3 node for the collection of audio information and an Edge for processing the audio and for the classification of birds with the AI module. Figure 1 shows an AI-IoT system schema, where the Edge is based on a Raspberry Pi 4B+ with 8 GB RAM. In the figure below, the processing module and the AI classification module are located in the Edge.

**Figure 2:**
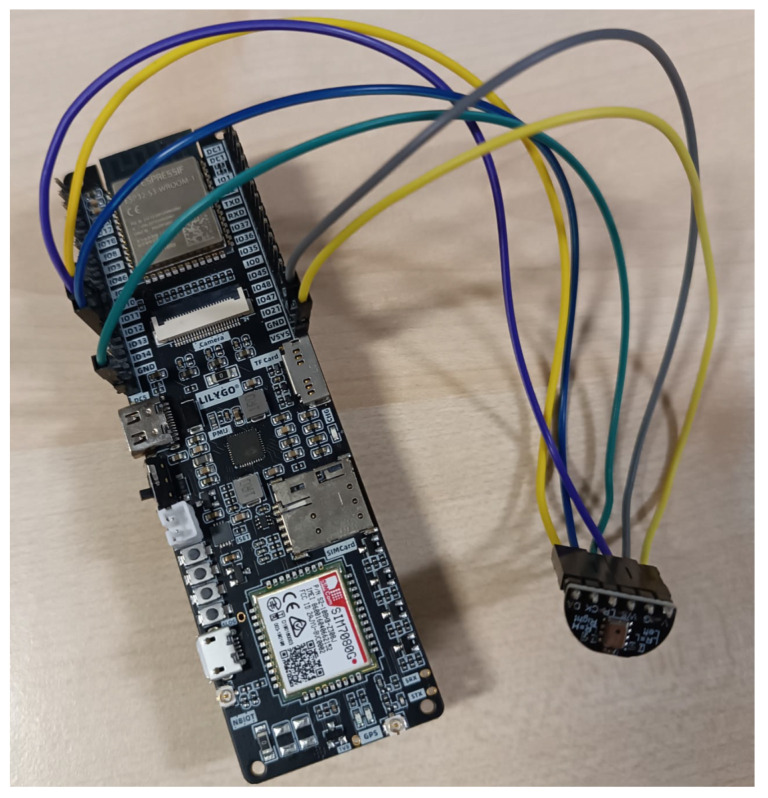
Photo of the ESP32-S3 MCU connected to the INMP441 microphone (Oakland, CA, USA).

The architecture of the IoT node for birdsong recording is based on a MEMS microphone (INMP441, CA/USA) and a microcontroller unit (MCU, here Lilygo T-SIM7080G based on ESP32-S3 WROOM-1-N16R8, Xinyuan/China). The MCU used has an embedded ESP32-S3 series of SoCs and an Xtensa dual-core 32-bit LX7 microprocessor (with up to 240 MHz). This MCU has up to 8 MB PSRAM and flash up to 16 MB. This MCU also has wireless communications with a WiFi interface (802.11 b/g/n), BLE (with embedded antenna), LTE-Cat-M and NB-IoT (with external antenna and SIM card connection), and different peripherals (I2C, SPI, UART, SDIO, I2S, CAN) (https://www.cnx-software.com/2023/03/04/esp32-s3-sim7080g-board-wifi-bluetooth-nb-iot-cat-m-gps/ (accessed on 23 October 2023)) [19]. It also allows for GNSS/GPS connection and the possibility of adding external storage with a microSD card. Figure 2 shows a photograph of the node with the microphone and the MCU. This MCU has been programmed with MQTT to publish audio, which will be collected into the Edge (Raspberry Pi 4B+ [6]) to collect audio and to do further processing.

### 2.2. Dataset and Augmentation Techniques

The dataset was obtained from the website of the xeno-canto Foundation and Naturalis Biodiversity Center (https://xeno-canto.org (accessed on 23 October 2023)). This foundation maintains a project collecting bird sounds from all over the world, and, from this website, we have selected 41 bird species that could be detected and identified within our system. These audio tracks collected for our dataset have been recorded with a sampling rate of 44,100 and 16-bit, and they have been selected as common European bird species, which, in many cases, are part of the environment of our cities. This dataset contains more than 1300 h of audio recordings in MP3 format (64 GB).

For the pre-processing stage, we have used 5 s audio chunks, processed as Mel-frequency cepstral coefficients (MFCC) spectrogram, establishing its parameters as the number of FFT, the window size, and the hop size to 1024, the number of Mel coefficients to 128, and the window type to Hanning. Each spectrogram has been saved in a portable network graphics format (named as 600 × 600 png image), which later will be used to train, validate, and test the different neural networks.

In Table 1, the second column shows the number of audio files obtained from the website above for each species of bird. The third column shows the number of spectrogram images (MFCC-based) obtained from the audio files for each species (here, each spectrogram takes a 5 s audio window).

**Figure 3:**
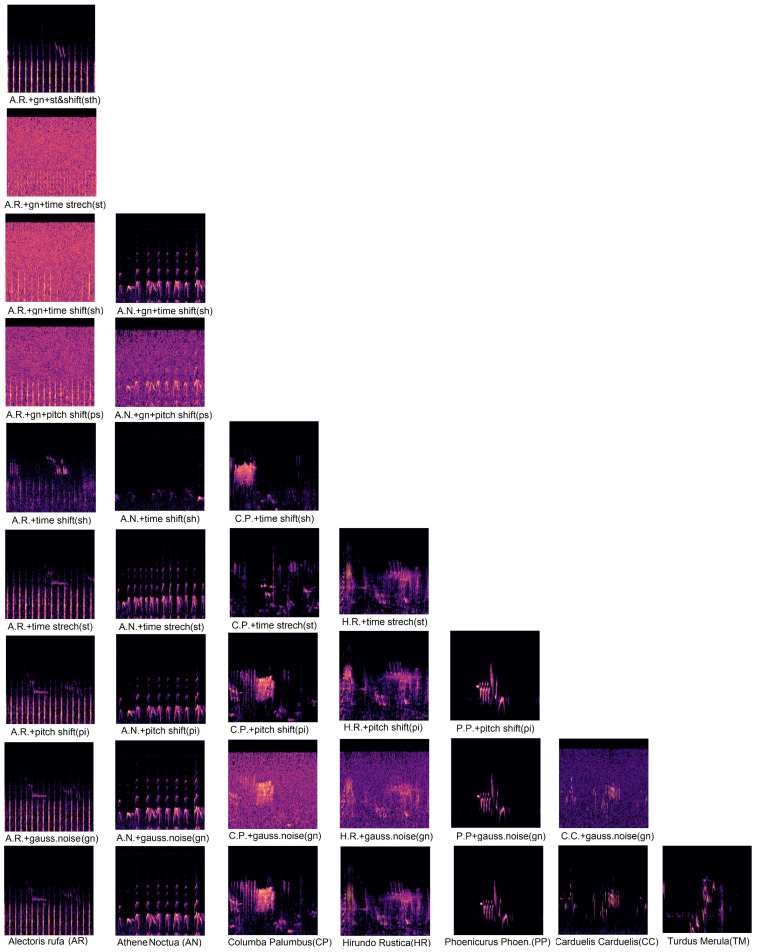
Examples of bird species with audio augmentation techniques using the criteria established.

In the fourth and fifth columns of Table 1, we have augmented the dataset with audio temporal augmentation techniques (such as white noise addition, time stretching, pitch shift, time shift, and combinations of these). They have been implemented using the libraries librosa and audiomentation in Python 3.8. At this point, we need to clarify that the algorithm used for the audio augmentation is based on the number of audio files, i.e., if the number of audio files is fewer than 200 files, the augmentation is done with “add noise”, “time stretching”, “pitch shifting”, “time shifting”, “combined add noise + time stretching”, “combined add noise + pitch shifting”, “combined add noise + time shifting”, and “combined add noise + stretching + time shifting”; if the number of audio files is between 200 and 400 files, the augmentation is done with “add noise”, “time stretching”, “pitch shifting”, “time shifting”, “combined add noise + time stretching”, “combined add noise + pitch shifting”, and “combined add noise + time shifting”; if the number of audio files is between 400 and 600 files, the augmentation is done with “add noise”, “time stretching”, “pitch shifting”, and “time shifting”; if the number of audio files is between 600 and 800 files, the augmentation is done with “add noise”, “pitch shifting”, and “time shifting”; if the number of audio files is between 800 and 1000 files, the augmentation is done with “add noise” and “pitch shifting”; if the number of audio files is between 1000 and 1500 files, the augmentation is done with “add noise”; and no technique is applied if the number of audio files is greater than 1500 files. With this augmentation, the dataset is increased considerably and it has the opportunity to be balanced. Figure 3 shows some examples of birdsong spectrograms with the corresponding enhancement techniques applied, paying attention to the criteria explained above. Apart from the possibility of enlarging the dataset, augmentation techniques can help to include some of the additional situations and problems that are not originally taken into account in the original dataset. For example, rain may introduce additional sounds not considered in a generally quieter environment, so Gaussian noise can simulate this more generic situation.

### 2.3. Different Neural Network Architectures

The hardware platform used for the training and experiments of the different CNN architectures was a desktop computer with 32 GB memory, Intel Core i9 7900X with 10 core and 20 thread CPU, 3.30 GHz frequency, and 3060 12 GB GPU. The operating system was Windows 10, 64-bit Professional. We also used Anaconda3, TensorFlow 2.10, and Python 3.8 as a deep learning platform.

For the implementation of the AI subsystem, we established a comparison of different options for the development of the AI classification module for the birds. The options considered here are based on the need for mobility for the devices used (which implies a low number of parameters for the inference), which can be single-board devices (SBC), such as Raspberry Pi 4B+ [6], or micro-controller units (MCU) with enough memory to contain the whole CNN weights file, such as ESP32-S3 [19].

For the adoption of this paradigm, we have selected, on one side, light-weight convolutional neural networks (CNNs) [23,24] based on architectures trained with Imagenet-weights, such as EfficientNet-B0 and -B4, and EfficientNetV2-Small and V2-B0, or MobileNet-v2 and -v3 Large and Small; and, on the other side, an ad hoc CNN architecture used to classify these spectrograms. This selection was based on the necessity to have a light-weight file to store the network weights for the inference stage into a SBC (such as Raspberry Pi 4B+) or even in a MCU (such as ESP32-S3 with 16 MB flash and 8 MB PSRAM, so we need to get some network lighter than 4 MB to enable this device to handle with it). Table 2 shows a comparison of these networks with the number of parameters and the weight of the resulting file (with flattened and dense layers in the output). Our purpose here is to find a proper deep convolutional neural network (DCNN) to be used in SBC or in MCU. According to this table, the Imagenet-based CNNs will be suitable for SBC, such as Raspberry Pi 4B+ with 4 GB, even the so-called DCNN 1, although it would fit properly in a MCU, such as ESP32-S3 with 8 MB PSRAM, but the operating system and the DCNN 1 would be too much for this MCU. The best option for the MCU is DCNN 2, which fits well and has enough RAM memory for further processing.

**Table 2:** Comparison of ConvNets according to the number of parameters.

ConvNet	No. of Parameters
EfficientNet v2S	20,383,881
EfficientNet B4	18,144,112
EfficientNet v2 B0	6,595,705
MobileNet v3L	4,925,033
MobileNet v2	4,829,545
EfficientNet B0	4,387,788
MobileNet v3S	2,096,345
Deep CNN 1 (Figure 4)	1,301,657
Deep CNN 2 (Figure 5)	331,745

Table 3, Table 4, Table 5, Table 6, Table 7, Table 8 and Table 9 show the structure and weights of each one of the studied ConvNets with the purpose to do transfer learning in the problem we are tackling.

## 3. Results and Discussion

In this section, we explain the process of training and validation with the normal dataset and the augmented dataset. From this process, we observed that data augmentation produced better results when no network layers were frozen during training. As we will see, with this transfer learning (using MobileNet V2, V3S, and V3L and EfficientNet B0, B4, V2S, and V2B0), we are able to achieve enough accuracy to distinguish the song of a specific bird specie, but we cannot achieve the accuracy in the state-of the-art result [17], which got an accuracy of 89.6% with 30 bird species with a multi-scale CNN, considering several options for the kernel in the Conv2D layer.

As we mentioned in Section 2, the original dataset was obtained directly from the xeno-canto webpage, and this project was developed thanks to the effort of ornithologists, but here the purpose is to use augmentation techniques to improve the performance of the predictions in future in-field applications.

### 3.1. Evaluation of Different Predictions

After the training and evaluation of the different networks, and as the original audio used was longer than 5 s required, the audio was divided into 5 s segments. We then computed the spectrogram for each segment and then exported them as PNG files. As we obtained different spectrogram images for each of the audio files, we then designed an algorithm for post-processing different spectrograms in the test stage, collecting together the files with the same prefix name, and in turn assembled the temporal sequence of spectrograms. Thus, by putting together the predictions in an array, we can vote for the preferred class, evaluate the correct one, and can therefore also compute the accuracy for this process. Figure 6 depicts a schema of this votation algorithm.

In Figure 7, we compare the average global accuracy obtained in the training process for the whole set of bird classes (41) and the use of this algorithm. After applying this procedure, we saw that accumulating consecutive predictions enables an increase in accuracy in the assessment of the class of a bird. Therefore, the IoT device will record for at least 15 s to enable this post-processing.

### 3.2. Performance Analysis

As a summary, we studied different CNN architectures and obtained the average performance metrics (precision, recall, F1-score) for the 42 bird classes, shown in Table 10 (the average support for all the classes is 5567.3). The average accuracy was also evaluated considering the voting algorithm explained for each network. As this table shows, the best performance is associated with the 5-layer deep CNN from 128 filters.

To obtain a reduced version of each model, we use Tensorflow-Lite (tflite) for integration into an SBC (such as Raspberry Pi) or an MCU (such as ESP32-S3). In our case, Table 11 shows the resulting weights of the files after the conversion process, without applying any quantization to avoid loss of accuracy.

In Table 11, we observe that the only model available for an MCU, such as ESP32-S3, is Deep CNN 2, with four convolutional layers, which is described in Figure 5. In this case, it should be noted that the rest of the PSRAM memory is needed for other OS processes (here it should be remembered that the ESP32-S3 can have 8 MB of PSRAM). The rest of the ConvNets could be used in SBCs, such as Raspberry Pi 4B+ with 4 or 8 GB, as they do not fit properly in the RAM of any low-power MCU.

Table 12 shows the average value of power consumption in several inference cycles for some of the studied CNNs in a Raspberry Pi 4B+. This performance measurement is related to the number of weights in the ConvNet.

## 4. Conclusions

In this work, we developed a monitoring system based on LTE-M/NB-IoT technologies that are 5G-based for massive machine type communications (mMTC), combined with an artificial intelligence module that allows the classification of up to 41 species of birds. For the development of this module, we selected different ConvNets, based on EfficientNet and MobileNet with ImageNet weights, and we compared their accuracy in training, validation, and testing.

**Figure 4:**
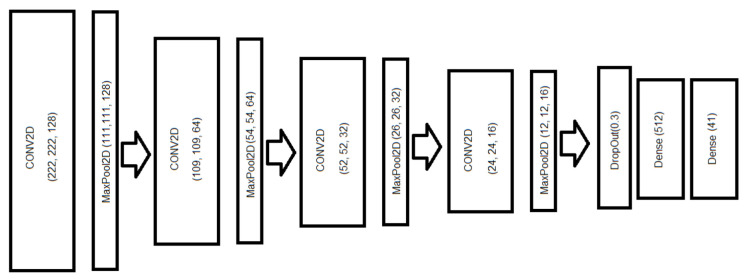
Structure of the proposed Deep CNN with four convolutional layers with MaxPool (with 128, 64, 32, and 16 filters, respectively).

**Figure 5:**
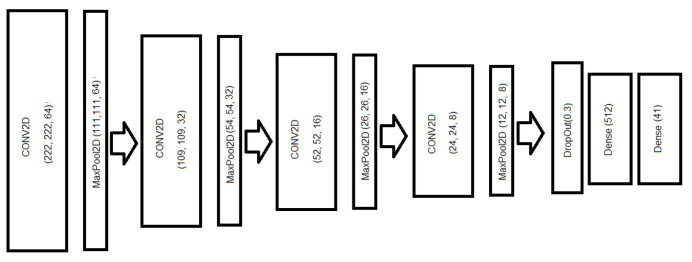
Structure of of the proposed Deep CNN with four convolutional layers with MaxPool (with 64, 32, 16, and 8 filters, respectively).

**Figure 6:**
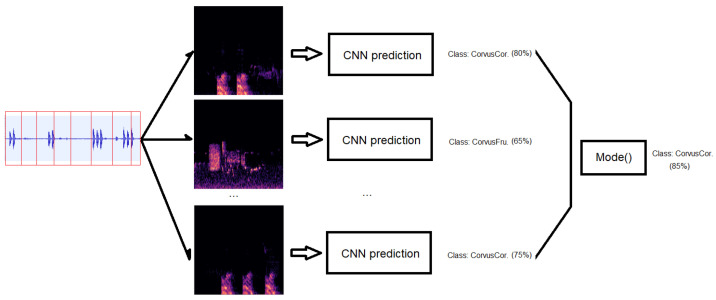
Votation algorithm schema for post-processing predictions to increase global accuracy for different predictions.

**Figure 7:**
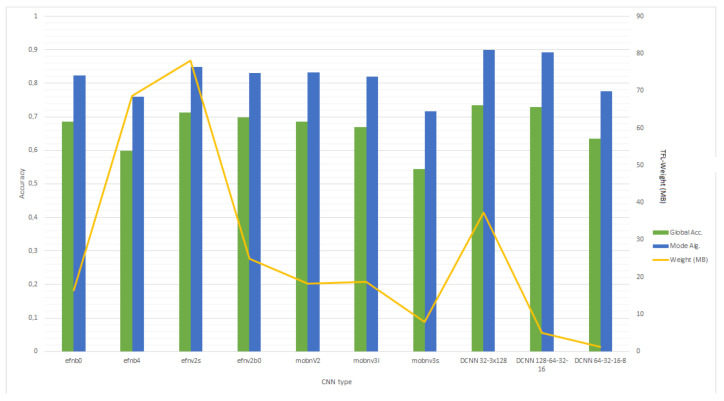
Comparison of the global accuracy in the test for the different networks selected and designed and the weight of the TensorFlow-Lite file.

In this study, we proposed the augmentation of the original dataset with the application of different signal processing techniques in the time domain to the original audio. For the augmented dataset, we obtained better accuracy and F1-score results than the original dataset. Also, we observed that for our augmented dataset, the best option in the EfficientNet family is EfficientNet v2 Small (71.4% accuracy), but it is heavier than the following network, which is EfficientNet v2 B0 (69.83% accuracy). For the MobileNet family, the best option is MobileNet v2, with 68.58% accuracy. The best option is, in all cases, 4-layer DCNN, which, depending on the structure of the number of filters, can have a higher weight. In this case, the most cost-effective option for the use in an SBC is DCNN 1 (72.95% accuracy), with a structure of 128-64-32-16 filters (shown in Figure 4, but the best option for an MCU is DCNN 2 (63.49% accuracy), with a structure of 64-32-16-8 filters (shown in Figure 5). Also, we propose a votation algorithm after the prediction step, which allows an improvement of around 22% in the global accuracy.

For further development, we will work with ConvNets to work directly with audio chunks. They will probably get lighter networks that will allow a direct application to MCUs with enough RAM.

## Figures and Tables

**Table 1:** Table with the different bird species considered, with the number of audio files collected, and the number of spectrograms images (each one has information from 5 s audio). We consider the normal audio set and the augmented audio set.

Species	No. Audio	No. Spectr. Samples	No. Augm. Audio	No. Augm. Spectr. Samples
*Alauda arvensis*	1447	22941	2897	45882
*Alectoris rufa*	103	1129	928	10161
*Apus apus*	48	689	433	6201
*Athene noctua*	286	5444	2033	38108
*Carduelis carduelis*	1079	16489	2161	33340
*Chloris chloris*	1065	14585	2133	29178
*Coccothraustes coccothraustes*	186	2098	1675	18882
*Columba palumbus*	498	6568	2492	32840
*Corvus cornix*	188	2206	1693	19862
*Corvus corone*	63	662	568	5958
*Corvus frugilegus*	42	685	379	6165
*Delichon urbicum*	89	2043	802	18387
*Dendrocopos major*	50	542	451	4878
*Emberiza citrinella*	1886	44867	1890	44867
*Erithacus rubecula*	2660	72123	2666	72123
*Fringilla coelebs*	3254	61676	3260	61676
*Garrulus glandarius*	213	3629	1492	25403
*Hirundo rustica*	753	13869	3014	55479
*Luscinia luscinia*	607	17789	2427	71471
*Motacilla alba*	217	2185	1520	15295
*Otus scops*	353	4574	2472	32018
*Parus major*	2858	41953	2864	41953
*Passer domesticus*	665	10419	2662	41676
*Passer montanus*	305	7694	2136	53858
*Perdix perdix*	93	1162	838	10458
*Phoenicurus ochruros*	679	11193	2718	44772
*Phoenicurus phoenicurus*	847	17890	2543	53670
*Phylloscopus collybita*	2475	48218	2481	48218
*Phylloscopus trochilus*	3394	111706	3401	111706
*Pica pica*	84	1137	757	10233
*Riparia riparia*	36	918	325	8262
*Sitta europaea*	534	6081	2672	30405
*Streptopelia decaocto*	519	6253	2597	31265
*Sturnus unicolor*	176	2382	1585	21438
*Sturnus vulgaris*	696	18964	2786	75856
*Troglodytes troglodytes*	2198	40168	2203	40168
*Turdus merula*	3195	127186	3202	127186
*Turdus philomelos*	3095	140193	3102	140193
*Turdus pilaris*	190	2739	1711	24659
*Turdus torquatus*	165	4366	1486	39294
*Tyto alba*	73	1515	658	13635
TOTAL	37392	899108	80366	1618681

**Table 3:** EfficientNet B0 network layers definition.

Layer (Type)	Output Shape	Param #
EfficientNet B0 (Functional)	(None, 7, 7, 1792)	4,049,571
GlobalAveragePooling2D	(None, 1792)	0
Dense	(None, 256)	327,680
Dropout (0.3)	(None, 256)	0
Dense	(None, 41)	10,537
Total params:		4,387,788 (16.74 MB)
Trainable params:		4,345,765 (16.58 MB)
Non-trainable params:		42,023 (164.16 KB)

**Table 4:** EfficientNet B4 network layers definition.

Layer (Type)	Output Shape	Param #
EfficientNet B4 (Functional)	(None, 7, 7, 1792)	17,673,823
GlobalAveragePooling2D	(None, 1792)	0
Dense	(None, 256)	458,752
Dropout (0.3)	(None, 256)	0
Dense	(None, 41)	10,537
Total params:		18,144,112 (69.21 MB)
Trainable params:		18,017,905 (68.73 MB)
Non-trainable params:		125,719 (489.09 KB)

**Table 5:** EfficientNet V2 small network layers definition.

Layer (Type)	Output Shape	Param #
EfficientNet V2—small (Functional)	(None, 7, 7, 1280)	20,331,360
GlobalAveragePooling2D	(None, 1280)	0
Dense	(None, 256)	458,752
Dropout (0.3)	(None, 256)	0
Dense	(None, 41)	52,521
Total params:		20,389,001 (77.78 MB)
Trainable params:		18,017,905 (68.73 MB)
Non-trainable params:		125,719 (489.09 KB)

**Table 6:** EfficientNet V2-B0 network layers definition.

Layer (Type)	Output Shape	Param #
EfficientNet V2—B0 (Functional)	(None, 7, 7, 1280)	25,919,312
GlobalAveragePooling2D	(None, 1280)	0
Dense	(None, 256)	327,680
Dropout (0.3)	(None, 256)	0
Dense	(None, 41)	10,537
Total params:		6,257,529 (18.78 MB)
Trainable params:		6,196,921 (18.73 MB)
Non-trainable params:		60,608 (489.09 KB)

**Table 7:** MobileNet V2 network layers definition.

Layer (Type)	Output Shape	Param #
MobileNet V2 1.00.224 (Functional)	(None, 7, 7, 1280)	2,257,984
Flatten (Flatten)	(None, 62,720)	0
Dropout (0.3)	(None, 62,720)	0
Dense	(None, 41)	2,571,561
Total params:		4,829,545 (18.42 MB)
Trainable params:		4,795,433 (18.29 MB)
Non-trainable params:		34,112 (133.25 KB)

**Table 8:** MobileNet V3 (small) network layers definition.

Layer (Type)	Output Shape	Param #
MobileNet V3—small (Functional)	(None, 7, 7, 576)	939,120
Flatten (Flatten)	(None, 28,224)	0
Dropout (0.3)	(None, 28,224)	0
Dense	(None, 41)	1,157,225
Total params:		2,096,345 (8.00 MB)
Trainable params:		2,084,233 (7.95 MB)
Non-trainable params:		12,112 (47.31 KB)

**Table 9:** MobileNet V3 (large) network layers definition.

Layer (Type)	Output Shape	Param #
MobileNet V3—large (Functional)	(None, 7, 7, 960)	2,996,352
Flatten (Flatten)	(None, 47,040)	0
Dropout (0.3)	(None, 47,040)	0
Dense	(None, 41)	1,928,681
Total params:		4,925,033 (18.79 MB)
Trainable params:		4,900,633 (18.69 MB)
Non-trainable params:		24,400 (95.31 KB)

**Table 10:** Average performance metrics of the main ConvNets used in this study (from transfer learning and DCNN).

	Avg Precision	Avg Recall	Avg F1-Score	Avg Accuracy (with Mode)
EfficientNet B0	0.6858	0.6934	0.6578	0.8235
EfficientNet B4	0.5983	0.5951	0.5664	0.7607
MobileNet v2	0.6549	0.6896	0.6582	0.8324
MobileNet v3s	0.5467	0.5517	0.5120	0.7172
DCNN 128	0.7056	0.7479	0.7174	0.8866
DCNN 64	0.6015	0.6520	0.6107	0.7731

**Table 11:** Comparison of the ConvNets converted to TfLite from Table 2.

Neural Network	TF-Lite Weight
EfficientNet v2—Small	78.1 MB
EfficientNet B4	68.6 MB
Deep CNN 0	37.2 MB
EfficientNet v2 B0	24.9 MB
MobileNet V3—Large	18.8 MB
MobileNet V2	18.2 MB
EfficientNet B0	16.5 MB
MobileNet V3—Small	8.0 MB
Deep CNN 1	5.0 MB
Deep CNN 2	1.3 MB

**Table 12:** Power consumption comparison of some of the studied ConvNets in a Raspberry Pi 4B+.

Neural Network	Average Power Consumption (W)
EfficientNet B4	4.78
EfficientNet B0	4.35
MobileNet V2	4.43
MobileNet V3—Small	4.32
Deep CNN 128	4.14
Deep CNN 64	4.04

## Data Availability

The original contributions presented in the study are included in the article, further inquiries can be directed to the corresponding author.
